# Short-term competition and long-term convergence between domestic and global rating agencies: Evidence from China

**DOI:** 10.1371/journal.pone.0232804

**Published:** 2020-05-21

**Authors:** Wei Tian, Xiangyun Zhou, Yixiang Tian, Wei Meng

**Affiliations:** School of Management and Economics, University of Electronic Science and Technology of China, Chengdu, China; Shandong University of Science and Technology, CHINA

## Abstract

This article adds to the existing literature on global rating agencies (GRAs, i.e., the S&P, Moody’s, and Fitch) and domestic rating agencies (DRAs). In our research, we introduce the reputation, rating cost and rating accuracy of rating agencies to improve the Hotelling model. According to the theoretical analysis and empirical tests, the results show that the open policy of the Chinese rating industry contributes to higher rating quality in the domestic bond market. This open policy leads to rating convergence between DRAs and GRAs from in the long term.

## Introduction

Ratings are important for the continuous development of the capital market. However, the phenomenon of rating inflation is obvious in Chinese corporate bonds. For default events in 2018, the ratings of default corporate bond were above AA. In fact, there are obvious differences between domestic ratings and foreign ratings. Most corporate bonds are rated AAA by DRAs (domestic rating agencies, DRAs) and given junk ratings by GRAs (global rating agencies, GRAs). Kou et al demonstrate that Chinese rating agencies have not acquired good market credibility; the quality of corporate bond ratings should be improved [1]. Huang et al find that the more defaults occur, the more credit rating agencies (CRAs) easily provide inflated ratings [2]. Therefore, it is of great importance to prevent inflated ratings and improve the rating quality of Chinese corporate bonds.

With the rapid opening up of the Chinese bond market, the Ministry of Finance of the People’s Republic of China, China’s Commerce Ministry and China’s Foreign Ministry issued the Early Harvest of the One Hundred-Day Plan for China-USA Economic Cooperation on May 12^th^, 2017. This plan announced that the Chinese rating industry promised foreign rating agencies that it would provide rating services in China, and GRAs could apply for credit permits before July 16^th^, 2017. The establishment of S&P (China) symbolized that the Chinese rating industry widely opened up to the world on January 28^th^, 2019.

Researchers have reported some expectations about the open policy for the rating industry. Some results show that the Chinese rating industry may increase competition, which will lead to reduced rating demand for DRAs in the short term. Sun finds that the big three CRAs have entered the rating market of China, which has led to a large adjustment in ratings and capital fluctuation [3]. Wang et al demonstrate that DRAs will lose considerable rating demand due to their poor credibility and low reputation in the fully open Chinese rating industry [4]. Others find that inclusive development among rating industries can avoid a massive concentration of domestic ratings and can improve the credibility of rating information [5]. Andre shows that Moody’s will adjust rating information when the S&P changes ratings, which leads to rating convergence between Moody’s and the S&P [6].

This study improves the Hotelling model and analyzes the competition among DRAs and GRAs from the short-term perspective and long-term perspective under the open policy of the rating industry. We utilize corporate bond data from the DRAs and GRAs to conduct empirical tests. Our research attempts to answer two question: First, can the open policy prevent rating inflation and improve rating quality? Second, can the open policy of the Chinese rating industry lead to rating convergence among the DRAs and GRAs?

In the remainder of the article, Section 2 provides a review of the relevant literature. Section 3 describes the construction of a new differential Hotelling model. In Section 4, we theoretically analyze the effect of the open policy of the rating industry on rating quality from the short-term perspective and the long-term perspective. In Section 5, we utilize panel ordered logit models to examine the research hypotheses proposed in the theoretical analysis. Section 6 briefly discusses the implications of our findings and prospects of future research.

## Review of the literature

Due to the good construction of American rating agencies, Japanese rating agencies and Korean rating agencies, many studies about the competition between GRAs and DRAs are mainly focused on these countries [7]. GRAs usually enter a domestic rating industry through the mergers and acquisitions of DRAs or by jointing with DRAs [8]. Due to increasing competition in the rating industry, GRAs may not contribute to the improvement of DRAs’ rating quality and often lead to rating divergence; namely, DRAs provide higher ratings [9]. Joe et al indicate that even if the foreign ownership of Korean rating agencies is increased, the rating quality steadily declines [10]. However, some researchers believe that DRAs attach more importance to the usage of qualitative information than GRAs. For example, Japanese rating agencies more carefully investigate and monitor Japanese corporations than GRAs; these agencies attempt to evaluate Japanese corporations in a comprehensive way [11]. Livingston et al consider Chinese corporate bond ratings and find that the rating standards of global-partnered rating agencies are more stringent, while the rating standards of DRAs are more lax [12].

Scholars have different opinions on the increased competition among rating agencies. Morkoetter et al suggest that the competition among CRAs makes each CRA provide high rating quality and more accurate information, which is good for investors [13]. Doherty claims that the entry of a new CRA can improve rating accuracy [14]. However, Manso finds that increasing competition among rating agencies may reduce rating pressure but enhance rating supervision [15].

There exists a close relationship among rating regulations, the reputation effect and rating quality. Alp indicates that strict rating supervision and investors’ dissatisfaction of inaccurate ratings induce rating agencies to provide more conservative ratings [16]. Dimitrov et al believe that after rating regulations have been enacted, rating agencies are more likely to provide lower ratings and more false alarms to protect their reputation; this downgrading in rating information is ineffective [17]. Becker and Milbourn think that the competition among rating agencies has an effect on their reputation, which leads to low ratings [18]. Sethuraman suggests that bond issuers voluntarily announce more qualitative information to help investors evaluate bond risk when rating agencies have low reputations [19]. Lugo et al show that due to the reputation effect, low-reputation rating agencies may issue ratings in accordance with high-reputation rating agencies, which leads to rating convergence among different rating agencies [5].

With the rapid opening up of the Chinese rating industry, competition among Chinese rating agencies and GRAs has increasingly become an important topic. For example, Livingston et al empirically compare the rating quality of Chinese rating agencies with the rating quality of global-partnered rating agencies [12]. In contrast to [12], we discuss the competition among DRAs and GRAs due to the open policy of the rating industry from the short-term perspective and the long-term perspective. There are a few theoretical studies about competition between DRAs and GRAs in China. In this article, we introduce the reputation, rating cost and rating accuracy of rating agencies to improve the Hotelling model. Based on the research hypothesis proposed by the Hotelling model, we utilize the ordered logit models to examine the regulatory results of the open policy. Finally, we demonstrate that this open policy can improve rating quality; corporate bond ratings provided by Chinese rating agencies and global-partnered rating agencies will converge due to the open rating policy.

## The differential Hotelling model

We develop a new model that builds on the Hotelling model [20], [21].

### Model assumptions

There exists one rating regulator, some DRAs and GRAs, and many rating buyers (bond issuers) in the market. The rating services provided by the DRAs and the GRAs have a strong substitute relationship, which causes differential competition between their rating prices and rating costs.

1. We assume that the rating demands of buyers are distributed uniformly from zero to one; the distribution density in the market is one. The rating demands of domestic ratings and global ratings are *q*_1_ and *q*_2_, respectively. We assume that the intrinsic utility of purchasing ratings is *u*_0_; the loss of utility that occurs when domestic ratings and global ratings are purchased is *q*_1_*t*_1_, *q*_2_*t*_2_, respectively; *t*_1_ is the unit loss that occurs when domestic ratings are purchased, for example, the implicit cost caused by low quality of ratings; *t*_2_ is the unit loss that occurs when global ratings are purchased, for example, the low rating demand caused by high global rating prices.
2. The rating prices, rating costs, rating demands, reputation, rating accuracy and profits of the DRAs and the GRAs are *p*_*i*_, *c*_*i*_, *q*_*i*_, *ρ*_*i*_, *ε*_*i*_, *π*_*i*_ (*i* = 1,2).
3. The reputation *ρ*_*i*_ can add ${\bar{u}}_{i}$ to the net utility of the rating buyers. Net utility is a monotonic increasing function of market share *α*_*i*_; namely ${\bar{u}}_{i}=\alpha_{i}\cdot \rho_{i}$ (*i* = 1,2).
4. The rating accuracy of the DRAs and the GRAs is *ε*_1_, *ε*_2_, respectively. If rating agencies provide high quality ratings, the net utility of rating buyers will increase ${\tilde{u}}_{i}=\alpha_{i}\cdot \varepsilon_{i}$ (*i* = 1,2).

### Construction of the improved Hotelling model

This paper improves the Hotelling model, by adding to some important factors such as the reputation, the rating cost and the rating accuracy of the rating agencies. We utilize this model to assess the effect of the mechanism of domestic and foreign dual ratings and the open policy of the rating industry on rating information from the short-term perspective and the long-term perspective.

The respective net utility of domestic ratings and global ratings for rating buyers is:

$$u_{1}=u_{0}+{\bar{u}}_{1}+{\tilde{u}}_{1}-p_{1}-q_{1}\cdot t_{1}$$ (1)

$$u_{2}=u_{0}+{\bar{u}}_{2}+{\tilde{u}}_{2}-p_{2}-q_{2}\cdot t_{2}$$ (2)

When *u*_1_ = *u*_2_, there is no difference between the domestic ratings and the global ratings purchased by rating buyers. Because the distribution density of the rating demands is one, the rating demands of buyers for domestic ratings and global ratings are *q*_1_ and *q*_2_, respectively, so we assume that *q*_1_+*q*_2_ = 1. Meanwhile, the market shares of the domestic ratings and global ratings are *α*_1_ and *α*_2_ for buyers, respectively, we assume that *α*_1_+*α*_2_ = 1. We can obtain the rating demands of DRAs *q*_1_ and GRAs *q*_2_ as follows:

$$q_{1}=\frac{1}{t_{1}+t_{2}}[t_{2}+\alpha_{1}\cdot (\rho_{1}+\varepsilon_{1})-\alpha_{2}\cdot (\rho_{2}+\varepsilon_{2})+p_{2}-p_{1}]$$ (3)

$$q_{2}=\frac{1}{t_{1}+t_{2}}[t_{1}-\alpha_{1}\cdot (\rho_{1}+\varepsilon_{1})+a_{2}\cdot (\rho_{2}+\varepsilon_{2})+p_{1}-p_{2}]$$ (4)

The profits of the DRAs and the GRAs are calculated as follows:

$$\pi_{1}=(p_{1}-c_{1})\cdot (\alpha_{1}+q_{1})$$ (5)

$$\pi_{2}=(p_{2}-c_{2})\cdot (\alpha_{2}+q_{2})$$ (6)

### Analysis of the factors in the improved Hotelling model

We utilize the improved Hotelling model to assess the impact of the rating accuracy and reputation of DRAs and GRAs on rating prices, rating demands and rating profits from the short-term perspective and the long-term perspective, respectively.

### Short-term game analysis

In the short term, the rating buyers will observe ratings provided by the DRAs. The market share *α*_2_ of global ratings is zero. The net utility of domestic ratings and global ratings for rating buyers are:

$$u_{1}=u_{0}+{\bar{u}}_{1}+{\tilde{u}}_{1}-p_{1}-q_{1}\cdot t_{1}$$ (7)

$$u_{2}=u_{0}-p_{2}-q_{2}\cdot t_{2}$$ (8)

Based on *q*_1_+*q*_2_ = 1, the respective rating demands of the DRAs *q*_1_ and the GRAs *q*_2_ are:

$$q_{1}=\frac{1}{t_{1}+t_{2}}[t_{2}+\alpha_{1}\cdot (\rho_{1}+\varepsilon_{1})+p_{2}-p_{1}]$$ (9)

$$q_{2}=\frac{1}{t_{1}+t_{2}}[t_{1}-\alpha_{1}\cdot (\rho_{1}+\varepsilon_{1})+p_{1}-p_{2}]$$ (10)

The respective profits of the DRAs and the GRAs are:

$$\pi_{1}=(p_{1}-c_{1})\cdot (\alpha_{1}+q_{1})=(p_{1}-c_{1})\cdot \frac{\left[t_{2}+\alpha_{1}\cdot (t_{1}+t_{2}+\rho_{1}+\varepsilon_{1})+p_{2}-p_{1}\right]}{t_{1}+t_{2}}$$ (11)

$$\pi_{2}=(p_{2}-c_{2})\cdot q_{2}=(p_{2}-c_{2})\cdot \frac{\left[t_{1}-\alpha_{1}\cdot (\rho_{1}+\varepsilon_{1})+p_{1}-p_{2}\right]}{t_{1}+t_{2}}$$ (12)

Based on the principle of the Hotelling model, when $\frac{\partial \pi_{1}}{\partial p_{1}}=0$ and $\frac{\partial \pi_{2}}{\partial p_{2}}=0$, we can obtain the short-term equilibrium solutions, which are provided in Table 1:

**Table 1:** Short-term equilibrium solutions of DRAs.

Rating prices and rating demand
$p_{1}^{*}=\frac{\left[2t_{2}+t_{1}+\alpha_{1}\cdot (\varepsilon_{1}+\rho_{1})+2\alpha_{1}\cdot (t_{1}+t_{2})+2c_{1}+c_{2}\right]}{3}$
$q_{1}^{*}=\frac{\left[t_{1}+2t_{2}+\alpha_{1}\cdot (\varepsilon_{1}+\rho_{1}-t_{1}-t_{2})+c_{2}-c_{1}\right]}{3(t_{1}+t_{2})}$
$\pi_{1}^{*}=\frac{\left[2t_{2}+t_{1}+\alpha_{1}\cdot (\varepsilon_{1}+\rho_{1})+2\alpha_{1}\cdot (t_{1}+t_{2})+2c_{1}+c_{2}]\cdot [t_{1}+2t_{2}+\alpha_{1}\cdot (\varepsilon_{1}+\rho_{1}-t_{1}-t_{2})+c_{2}-c_{1}\right]}{9(t_{1}+t_{2})}$

According to Table 1, by calculating the derivatives of price and demand, we can obtain dp1*dε1>0 dp1*dρ1>0. These results demonstrate that rating accuracy and reputation have positive effects on the rating price for DRAs. When GRAs enter the rating market over a short period of time, the DRAs will increase their prices due to rating competition. According to dq1*dε1>0, dq1*dρ1>0, rating accuracy and reputation have positive effects on rating demand of the DRAs. The results show that high rating quality and high reputation can increase the rating demands for the DRAs in the short term. Due to the increasing rating accuracy and reputation of the DRAs, the rating prices and rating demands increase. According to $\pi_{1}^{*}=p_{1}^{*}\cdot q_{1}^{*}$, dπ1*dε1>0, dπ1*dρ1>0, we can conclude that rating profits of the DRAs also increase. Competition among DRAs and GRAs can effectively push DRAs to improve their rating quality and reputation and thus increase their profit in the short term.

According to Table 2, by calculating the derivatives of price and demand, we can obtain dp2*dε1<0, dp2*dρ1<0, which demonstrates that the rating accuracy and the reputation of the DRAs have negative effects on the rating price of the GRAs. Due to dq2*dε1<0, dq2*dρ1<0, the rating accuracy and the reputation of the DRAs also have negative effects on the rating demands for GRAs. According to $\pi_{2}^{*}=p_{2}^{*}\cdot q_{2}^{*}$, dπ2*dε1<0, dπ2*dρ1<0, we can conclude that the rating profits of the GRAs decrease with high rating accuracy and high reputation of the DRAs.

**Table 2:** Short-term equilibrium solutions of GRAs.

Rating prices and rating demand
$p_{2}^{*}=\frac{\left[2t_{1}+t_{2}-\alpha_{1}\cdot (\varepsilon_{1}+\rho_{1})+\alpha_{1}\cdot (t_{1}+t_{2})+c_{1}+2c_{2}\right]}{3}$
$q_{2}^{*}=\frac{\left[2t_{1}+t_{2}-\alpha_{1}\cdot (\varepsilon_{1}+\rho_{1}-t_{1}-t_{2})+c_{1}-c_{2}\right]}{3(t_{1}+t_{2})}$
$\pi_{2}^{*}=\frac{\left[2t_{1}+t_{2}-\alpha_{1}\cdot (\varepsilon_{1}+\rho_{1})+\alpha_{1}\cdot (t_{1}+t_{2})+c_{1}+2c_{2}]\cdot [2t_{1}+t_{2}-\alpha_{1}\cdot (\varepsilon_{1}+\rho_{1}-t_{1}-t_{2})+c_{1}-c_{2}\right]}{9\cdot {\left(t_{1}+t_{2}\right)}^{}}$

The above results illustrate that the short term competition among DRAs and GRAs can inspire DRAs to improve their rating quality and reputation to increase their profits. However, due to the high rating quality and high reputation of DRAs, GRAs will obtain less rating profit. We show that it is difficult for the rating accuracy of DRAs and GRAs to reach convergence in the short term.

### Long-term game analysis

In the long term, GRAs will obtain market share *α*_2_. It is effective for DRAs to learn more advanced techniques and methods used to calculate credit ratings from GRAs. The open policy of the rating industry contributes to rating learning among DRAs and GRAs. Under this policy, we assume that rating accuracy of the DRAs and the GRAs can achieve convergence.

The respective net utility of domestic ratings and global ratings for rating buyers are:

$$u_{1}=u_{0}+{\bar{u}}_{1}+{\tilde{u}}_{1}-p_{1}-q_{1}\cdot t_{1}$$ (13)

$$u_{2}=u_{0}+{\bar{u}}_{2}+{\tilde{u}}_{2}-p_{2}-q_{2}\cdot t_{2}$$ (14)

Based on *q*_1_+*q*_2_ = 1, the respective rating demands of DRAs *q*_1_ and GRAs *q*_2_ are:

$$q_{1}=\frac{1}{t_{1}+t_{2}}[t_{2}+\alpha_{1}\cdot (\varepsilon_{1}+\rho_{1})-\alpha_{2}\cdot (\varepsilon_{2}+\rho_{2})+p_{2}-p_{1}]$$ (15)

$$q_{2}=\frac{1}{t_{1}+t_{2}}[t_{1}-\alpha_{1}\cdot (\rho_{1}+\varepsilon_{1})+\alpha_{2}\cdot (\rho_{2}+\varepsilon_{2})+p_{1}-p_{2}]$$ (16)

The respective profits of DRAs and GRAs are:

$$\pi_{1}=(p_{1}-c_{1})\cdot (\alpha_{1}+q_{1})=(p_{1}-c_{1})\cdot \frac{\left[t_{2}+\alpha_{1}\cdot (t_{1}+t_{2}+\rho_{1}+\varepsilon_{1})-\alpha_{2}\cdot (\rho_{2}+\varepsilon_{2})+p_{2}-p_{1}\right]}{t_{1}+t_{2}}$$ (17)

$$\pi_{2}=(p_{2}-c_{2})\cdot (\alpha_{2}+q_{2})=(p_{2}-c_{2})\cdot \frac{\left[t_{1}-\alpha_{1}\cdot (\rho_{1}+\varepsilon_{1})+\alpha_{2}\cdot (t_{1}+t_{2}+\rho_{2}+\varepsilon_{2})+p_{1}-p_{2}\right]}{t_{1}+t_{2}}$$ (18)

When $\frac{\partial \pi_{1}}{\partial p_{1}}=0$ and $\frac{\partial \pi_{2}}{\partial p_{2}}=0$, we can obtain the long-term equilibrium solutions under the open policy of the rating industry:

From Table 3, we know that ∂p1*∂ε1>0, ∂p1*∂ρ1>0. The results show that the rating accuracy and the reputation of DRAs have positive effects on the rating prices of DRAs. According to ∂q1*∂ε1>0, and ∂q1*∂ρ1>0, the rating accuracy and the reputation of DRAs also have positive effects on the rating demand of DRAs. Due to ∂π1*∂ε1>0, and ∂π1*∂ρ1>0, the results show that the rating accuracy and the reputation of DRAs have positive effects on the rating profit of DRAs.

**Table 3:** Long-term equilibrium solutions of DRAs.

Rating price and rating demand
$p_{1}^{*}=\frac{\left[t_{1}+2t_{2}+(\alpha_{2}+2\alpha_{1})\cdot (t_{1}+t_{2})+\alpha_{1}\cdot (\varepsilon_{1}+\rho_{1})-\alpha_{2}\cdot (\varepsilon_{2}+\rho_{2})+2c_{1}+c_{2}\right]}{3}$
$q_{1}^{*}=\frac{\left[t_{1}+2t_{2}+\alpha_{1}\cdot (\varepsilon_{1}+\rho_{1})-\alpha_{2}\cdot (\varepsilon_{2}+\rho_{2})-(\alpha_{1}-\alpha_{2})\cdot (t_{1}+t_{2})+c_{2}-c_{1}\right]}{3(t_{1}+t_{2})}$
$\pi_{1}^{*}=\frac{\left[(1+\alpha_{1})\cdot t_{1}+(2-\alpha_{2})\cdot t_{2}+\alpha_{1}\cdot (\varepsilon_{1}+\rho_{1})-\alpha_{2}\cdot (\varepsilon_{2}+\rho_{2})+2c_{1}+c_{2}]\cdot [t_{1}+\alpha_{1}\cdot (2t_{1}-t_{2}+\varepsilon_{1}+\rho_{1})-\alpha_{2}\cdot (t_{2}+\varepsilon_{2}+\rho_{2})+c_{2}-c_{1}\right]}{9{\left(t_{1}+t_{2}\right)}^{}}$

From Table 4, we know that ∂p2*∂ε2>0, ∂p2*∂ρ2>0. The results show that the rating accuracy and the reputation of GRAs have positive effects on the rating prices of GRAs. According to ∂q2*∂ε2>0, ∂q2*∂ρ2>0, the rating accuracy and the reputation of GRAs also have positive effects on rating demand for GRAs. Due to ∂π2*∂ε2>0, ∂π2*∂ρ2>0, the results show that the rating accuracy and the reputation of GRAs have positive effects on the profit of GRAs.

**Table 4:** Long-term equilibrium solutions of GRAs.

Rating price and rating demand
$p_{2}^{*}=\frac{\left[t_{2}+2t_{1}+(\alpha_{1}+2\alpha_{2})\cdot (t_{1}+t_{2})-\alpha_{1}\cdot (\varepsilon_{1}+\rho_{1})+\alpha_{2}\cdot (\varepsilon_{2}+\rho_{2})+2c_{2}+c_{1}\right]}{3}$
$q_{1}^{*}=\frac{\left[2t_{1}+t_{2}-\alpha_{1}\cdot (\varepsilon_{1}+\rho_{1})+\alpha_{2}\cdot (\varepsilon_{2}+\rho_{2})-(\alpha_{1}-\alpha_{2})\cdot (t_{1}+t_{2})+c_{1}-c_{2}\right]}{3(t_{1}+t_{2})}$
$\pi_{2}^{*}=\frac{\left[t_{2}+2t_{1}+(\alpha_{1}+2\alpha_{2})\cdot (t_{1}+t_{2})-\alpha_{1}\cdot (\varepsilon_{1}+\rho_{1})+\alpha_{2}\cdot (\varepsilon_{2}+\rho_{2})+2c_{2}+c_{1}]\cdot [2t_{1}+t_{2}-\alpha_{1}\cdot (\varepsilon_{1}+\rho_{1})+\alpha_{2}\cdot (\varepsilon_{2}+\rho_{2})-(\alpha_{1}-\alpha_{2})\cdot (t_{1}+t_{2})+c_{1}-c_{2}\right]}{9{\left(t_{1}+t_{2}\right)}^{}}$

Based on the above results, we find that the two types of rating agencies will improve rating accuracy and their reputation to increase their profit under a long-term open policy. This policy is effective for improving the rating quality of DRAs and GRAs who will seek to increase their profit. Furthermore, this policy can promote rating convergence through long-term competition among DRAs and GRAs.

Research hypothesis: When long-term competition occurs among DRAs and GRAs, it is good for DRAs and GRAs to improve their rating quality and reach rating convergence under the open policy.

## Empirical analysis

Due to the lack of rating data provided by the GRAs, in this article, we replace GRAs with global-partnered rating agencies. On the basis of [12], we examine the research hypothesis and discuss the effects of competition among DRAs and global-partnered ratings agencies on Chinese corporate bond ratings. In contrast to [12], we add relevant variables to describe the reputation effect, rating cost and rating accuracy of rating agencies. In addition, we analyze the regulatory effect of the open policy of the Chinese rating industry. Furthermore, we compare the changes in the corporate bond ratings issued by the DRAs and the global-partnered ratings agencies before and after introducing the open policy. In addition, we discuss whether the ratings of the DRAs and the global-partnered ratings agencies achieve convergence.

Table 5 lists the cooperation between eight Chinese major rating agencies and the GRAs. Moody’s has had 49% ownership in Chengxin_Moody since 2006. Fitch has had 49% ownership in Lianhe_Fithch since 2007. Brilliance has had technical partnership with the S&P since 2008. The other rating agencies, DRAs, do not cooperate with GRAs.

**Table 5:** List of major Chinese credit rating agencies.

Name	Global partner
Brilliance	S&P
Chengxin_Moody	Moody’s
Lianhe_Fitch	Fitch
Dagong	None
Chengxin	None
Jincheng	None
Lianhe	None
Pengyuan	None

Source: [12].

### Description of the data and sample

We collected all data from the Wind database. It has been only a short time since the open policy of the Chinese rating industry was enacted, we replace the GRAs that do not have any ratings data with the global-partnered ratings agencies. We use a sample period from 2003 to 2018. The dependent variables are ordinal variables, Chinese corporate bond ratings, and are given values ranging from 1 (for AAA) to 22 (for D). Rating downgrades and rating upgrades are positive numbers indicating the difference between the nearest rating scores and the previous rating scores.

The explanatory variables are eight dummy variables, including variables representing the open policy of the rating industry and different rating agencies. To assess the open policy of the Chinese rating industry, if rating agencies provided corporate bond ratings after July 16th, 2017, this variable will equal one; otherwise, it will be zero. There are many differences among rating agencies in term of reputation, rating costs and rating accuracy. We utilize seven dummy variables to indicate that different CRAs, Brilliance, Lian_Fitch, Dagong, Chengxin, Jincheng, Lianhe and Pengyuan. If ratings are issued by one of the DRAs, this variable will be equal one.

As the prior literature shows, asset-liability ratio, profitability, solvency, liquidity and industry classification are important factors for corporate bond ratings. Therefore, we select a series of relevant indicators such as asset-liability ratio, operating profit margin, return on total assets, EBITDA/total revenue, short term debt ratio, debt with interest/invested capital, currency/short term debt, currency/total debt, current ratio, quick ratio and industry classification as control variables [22], [23].

Based on [5], we utilize the market share of the different rating agencies to describe the reputation effect. A larger market share responds to a higher reputation effect. Table 6 shows that Pengyun has the highest market share overall; the second is Dagong. Chengxin_Moody, Brilliance and Linahe_Fithch are the third, fourth and sixth, respectively. Jincheng has the smallest market share. We introduce two dummy variables to account for the global-partnered rating agencies and different rating agencies and to describe the reputation effect.

**Table 6:** Market shares of different rating agencies.

Year	Brilliance	Chengxin_Moody	Lianhe_Fitch	Dagong	Chengxin	Jincheng	Lianhe	Pengyuan
2003	0	2	0	2	0	0	0	0
2004	0	0	2	2	0	0	0	0
2005	2	6	7	0	0	0	0	0
2006	2	27	2	8	0	0	0	0
2007	7	42	16	8	0	0	0	0
2008	11	41	22	24	0	0	0	0
2009	16	66	43	25	4	0	0	1
2010	31	79	45	50	4	0	6	15
2011	57	112	76	87	13	0	14	83
2012	117	218	123	226	33	3	26	352
2013	201	287	242	352	44	31	32	574
2014	278	490	315	467	67	119	64	967
2015	463	588	456	650	222	294	287	1130
2016	638	702	568	975	691	417	771	1546
2017	804	797	645	1043	890	522	958	1674
2018	850	828	678	1088	1114	564	1127	1691
Total	3477	4285	3240	5007	3082	1950	3285	8033

Source: the Wind database.

### Ordered logit model

The credit rating variable is an ordinal and qualitative variable. The traditional linear transformation of ratings will not be hold here; because of the boundaries of rating symbols, this type of transformation will lead to errors when large samples are employed. We utilize the discrete choice model to avoid this problem. This model can subject each rating symbol to interval truncation. The model employs both a continuous unobservable potential variable $y_{it}^{*}$ and an observable rating variable *y*_*it*_ [24]. The ordered logit model is:

$$y_{it}^{*}=x_{it}^{'}\beta +w_{i}^{'}\lambda +\alpha_{i}+\varepsilon_{it}\;i=1,\ldots ,N\;t=1,\ldots ,T$$ (19)

In this model, $x_{it}^{'}$ is an independent variable that changes over time and for each individual object; $w_{i}^{'}$ is a dummy variable, indicating whether the open policy discussed in this article has been enacted; *α*_*i*_ represents the heterogeneity of corporate bonds; and the distribution of residual *ε*_*it*_ is independent of time and individual objects.

The relationship between the observable variable *y*_*it*_ and the continuous unobservable potential variable $y_{it}^{*}$ in this model is as follows:

$$y_{it}=\{\begin{array}{ccc} AAA & if & y_{it}^{*}\in \tau_{1}\equiv \left(-\infty ,c_{1}\right]\\ AA^{+} & if & y_{it}^{*}\in \tau_{2}\equiv \left(c_{1},c_{2}\right]\\ AA & if & y_{it}^{*}\in \tau_{3}\equiv \left(c_{2},c_{3}\right]\\ \cdots & \cdots & \cdots \\ CC & if & y_{it}^{*}\in \tau_{21}\equiv \left(c_{20},c_{21}\right]\\ C & if & y_{it}^{*}\in \tau_{22}\equiv \left(c_{21},c_{22}\right]\\ D & if & y_{it}^{*}\in \tau_{23}\equiv \left(c_{22},+\infty \right) \end{array}$$ (20)

The number of truncated points *c*_1_,*c*_2_,…,*c*_22_ is determined by the number of observable variables *y*_*it*_. We can estimate the values of each truncated point through the maximum likelihood function. According to the properties of the ordered logit model, the conditional distribution of observable variable *y*_*it*_ is determined by the distribution of residual *ε*_*it*_. We let *s* = [*s*_1_,*s*_2_,…,*s*_*j*_,…,*s*_23_]' = [*AAA*,*AA*^+^,…,*C*,*D*]', and the probability of *y*_*it*_ in the state *s*_*j*_ is:

$$\mathrm{Pr}(y_{it}=s_{j}|x_{it}^{'},w_{i}^{'})=\mathrm{Pr}(y_{it}^{*}\in \tau_{i}|x_{it}^{'},w_{i}^{'})=\mathrm{Pr}(x_{it}^{'}\beta +w_{i}^{'}\lambda +\alpha_{i}+\varepsilon_{it}\in \tau_{i}|x_{it}^{'},w_{i}^{'})$$ (21)

$$\mathrm{Pr}(y_{it}=s_{j}|x_{it}^{'},w_{i}^{'})=\{\begin{array}{c} p(x_{it}^{'}\beta +w_{i}^{'}\lambda +\alpha_{i}+\varepsilon_{it}\leq c_{1}|x_{it}^{'},w_{i}^{'},\alpha_{i}),j=1\\ p(c_{j-1}<x_{it}^{'}\beta +w_{i}^{'}\lambda +\alpha_{i}+\varepsilon_{it}\leq c_{j}|x_{it}^{'},w_{i}^{'},\alpha_{i}),1<j<22\\ p(c_{22}<x_{it}^{'}\beta +w_{i}^{'}\lambda +\alpha_{i}+\varepsilon_{it}|x_{it}^{'},w_{i}^{'},\alpha_{i}),j=22 \end{array}$$ (22)

If the distribution of residual *ε*_*it*_ satisfies the logistic cumulative distributed function [25]; namely:

$$F\left(\varepsilon_{it}|x_{it}^{'},w_{i}^{'}\right)=F\left(\varepsilon_{it}\right)=\frac{e^{\varepsilon }}{1+e^{\varepsilon }}=\Lambda \left(\varepsilon_{it}\right)$$ (23)

Eq (21) can be written as follows:

$$\mathrm{Pr}\left(y_{it}=s_{j}|x_{it}^{'},w_{i}^{'}\right)=\{\begin{array}{c} \Lambda \left(c_{1}-x_{it}^{'}\beta -w_{i}^{'}\lambda -\alpha_{i}\right),j=1\\ \Lambda \left(c_{j-1}-x_{it}^{'}\beta -w_{i}^{'}\lambda -\alpha_{i}\right)-\Lambda \left(c_{j}-x_{it}^{'}\beta -w_{i}^{'}\lambda -\alpha_{i}\right)\\ 1-\Lambda \left(c_{j}-x_{it}^{'}\beta -w_{i}^{'}\lambda -\alpha_{i}\right),j=22 \end{array},1<j<22$$ (24)

*F*(•) is the logistic cumulative distributed function of *ε*_*it*_; *s*_*j*_ is the state of observing the corporate bond rating in time *t*; and *τ*_*i*_ represents the strictly increasing truncated points.

Some researchers utilize the ordered logit model to study the effect of the new entrance of one rating agency on ratings [26], [27]. Bakalyar and Galil employ the ordered logit model and show that when Moody’s provides higher ratings, the S&P also issues inflated ratings, which leads to rating shopping [28]. Therefore, we examine the research hypotheses using the ordered logit model.

### Empirical results

Table 7 reports the changes in corporate bond ratings. For the Column 1 model and Column 2 model, which is based on [12], we choose Chengxin_Moody as the base case because it rates more bonds in the sample than any other rating agencies.

**Table 7:** Regression results of corporate bond ratings.

Variables	Ordered logit (1)	Ordered logit (2)
Operating profit margin	-0.000012*** (2.55e-06)	-0.00001*** (2.49e-06)***
Return on total assets	-0.007*** (0.002)	-0.008*** (0.002)
EBITDA/total revenue	0.0001*** (0.00002)	0.0001*** (0.00002)
Current ratio	-0.002 (0.002)	-0.001 (0.002)
Quick ratio	0.068*** (0.007)	0.066*** (0.007)
Asset-liability ratio	-0.009*** (0.001)	-0.009*** (0.001)
Short term debt ratio	0.003*** (0.001)	0.003*** (0.001)
Debt with interest/invested capital	-0.009*** (0.001)	-0.009*** (0.001)
Currency/short term debt	-0.125*** (0.015)	-0.127*** (0.015)
Currency/total debt	-0.592*** (0.058)	-0.590*** (0.058)
The open policy of the rating industry		0.430*** (0.024)
Brilliance	0.762*** (0.043)	0.791*** (0.043)
Lianhe_Fitch	0.471*** (0.045)	0.476*** (0.045)
Dagong	0.432*** (0.041)	0.442*** (0.041)
Chengxin	0.086** (0.047)	0.169*** (0.047)
Jincheng	1.318*** (0.054)	1.367*** (0.055)
Lianhe	0.743*** (0.047)	0.816*** (0.047)
Pengyuan	-1.263*** (0.037)	1.274*** (0.037)
Industry dummies	Yes	Yes
*C*_*1*_	-0.138 (0.052)	-0.245 (0.053)
*C*_*2*_	1.164 (0.052)	1.078 (0.052)
*C*_*3*_	5.494 (0.073)	5.422 (0.073)
*C*_*4*_	6.109 (0.086)	6.036 (0.086)
*C*_*5*_	6.214 (0.089)	6.141 (0.089)
*C*_*6*_	6.439 (0.096)	6.365 (0.096)
*C*_*7*_	6.486 (0.098)	6.413 (0.098)
*C*_*8*_	6.558 (0.100)	6.484 (0.101)
*C*_*9*_	6.643 (0.104)	6.570 (0.104)
*C*_*10*_	6.676 (0.105)	6.603 (0.105)
*C*_*11*_	6.782 (0.109)	6.708 (0.109)
*C*_*12*_	7.022 (0.120)	6.948 (0.120)
*C*_*13*_	7.322 (0.136)	7.248 (0.136)
*C*_*14*_	7.371 (0.139)	7.297 (0.139)
*C*_*15*_	7.476 (0.146)	7.403 (0.146)
*C*_*16*_	7.855 (0.172)	7.781 (0.172)

Table 7 reports that the numbers in the parentheses are standard errors.

*, **, *** denote that the coefficient is statistically significant at the 10%, 5%, 1% levels respectively.

In the Column 1 model, all of the financial indicators are significant determinants of corporate bond ratings. Without considering the open policy, the coefficient of Pengyuan is significantly negative. The coefficients of other CRAs are significantly positive. The results show that there are differences in the rating standards of DRAs and GRAs. The rating standard of Pengyuan is more lax, while the rating standards of other CRAs are more stringent.

In the Column 2 model, we introduce the open policy. The coefficient of the open policy is significantly positive, which demonstrates that this policy has a positive effect on rating scores. Competition among the DRAs and global-partnered rating agencies can improve rating standards. Meanwhile, the coefficients of all of the CRAs are positive and significant at the 1% level. The rating standards of the DRAs and the global-partnered rating agencies are more stringent under the open policy.

Due to the rating learning effect and the regulatory effect, the results are obviously different from previous studies [12] that do not consider the open policy. The results show that competition among global-partner CRAs and DRAs contributes to higher rating quality under the open policy.

Furthermore, we analyze the effect of the policy on rating downgrades and rating upgrades. Table 8 reports the empirical results of rating downgrades. In the Column 1 model, the operating profit margin, current ratio and quick ratio are significant determinants of rating downgrades. Without considering the open policy, the DRAs and the GRAs have obvious differences in rating standards. Lianhe_Fitch and Jincheng have a negative effect on rating downgrades, while other CRAs have a positive effect on rating upgrades.

**Table 8:** Regression results of rating downgrades.

Variables	Ordered logit (1)	Ordered logit (2)
Operating profit margin	0.012** (0.005)	0.006 (0.009)
Return on total assets	-0.016 (0.020)	-0.014 (0.020)
EBITDA/total revenue	0.001 (0.002)	-0.004 (0.009)
Current ratio	-2.137*** (0.483)	-1.135*** (0.431)
Quick ratio	2.247*** (0.454)	0.834* (0.441)
Asset-liability ratio	0.013 (0.014)	-0.003 (0.015)
Short term debt ratio	0.001 (0.008)	-0.004 (0.008)
Debt with interest/invested capital	0.0005 (0.011)	-0.004 (0.011)
Currency/short term debt	-1.323 (0.937)	0.250 (0.881)
Currency/total debt	2.283 (1.433)	-0.340 (1.381)
The open policy of the rating industry		1.990*** (0.263)
Brilliance	0.385 (1.109)	0.566 (1.011)
Lianhe_Fitch	-0.647 (0.749)	0.614 (0.742)
Dagong	0.548 (0.551)	0.190 (0.564)
Chengxin	0.602 (0.639)	0.427 (0.644)
Jincheng	-0.049 (0.759)	0.079 (0.778)
Lianhe	0.843 (0.513)	0.682 (0.523)
Pengyuan	0.686 (0.505)	0.333 (0.514)
Industry dummies	Yes	Yes
*C*_*1*_	1.956 (0.962)	0.447 (1.061)
*C*_*2*_	2.494 (0.964)	1.078 (1.060)
*C*_*3*_	2.952 (0.966)	1.624 (1.061)
*C*_*4*_	3.278 (0.970)	2.008 (1.063)
*C*_*5*_	3.540 (0.973)	2.319 (1.066)
*C*_*6*_	4.045 (0.979)	2.894 (1.070)
*C*_*7*_	4.468 (0.984)	3.350 (1.073)
*C*_*8*_	5.000 (0.992)	3.903 (1.079)
*C*_*9*_	5.360 (1.002)	4.270 (1.087)
*C*_*10*_	5.945 (1.010)	4.505 (1.095)
*C*_*11*_	5.786 (1.018)	4.695 (1.102)
*C*_*12*_	6.314 (1.050)	5.225 (1.130)
*C*_*13*_	6.501 (1.065)	5.417 (1.144)

Table 8 reports the numbers in the parentheses are standard errors.

*, **, *** denote that the coefficient is statistically significant at the 10%, 5%, 1% levels respectively.

In the Column 2 model, we introduce the regulatory effect of the open policy of the rating industry on rating downgrades. The coefficients of the open policy of the rating industry are positive and significant at the 1% level. This result shows that increasing competition among DRAs and GRAs can lead to lower ratings. Meanwhile, after the open policy, the coefficients of all CRAs are positive but not significant. The findings show that the open policy leads to rating convergence among DRAs and GRAs in terms of rating downgrades.

Table 9 shows the results of rating upgrades. In the Column 1 model, return on assets, debt with interest/invested capital and currency/total debt are significant determinants of rating upgrades. Without considering the open policy, there are some differences in the rating standards of DRAs and GRAs. The coefficient of Chengxin is negative, while the coefficients of other CRAs are positive. This result indicates that rating differences exist among competing CRAs without regulatory effects.

**Table 9:** Regression results of rating upgrades.

Variables	Ordered logit (1)	Ordered logit (2)
Operating profit margin	0.0001 (0.0001)	0.0001 (0.0001)
Return on total assets	0.022** (0.012)	0.022* (0.012)
EBITDA/total revenue	0.00003 (0.00002)	0.00003 (0.00003)
Current ratio	-0.001 (0.005)	-0.001 (0.005)
Quick ratio	-0.003 (0.029)	-0.006 (0.029)
Asset-liability ratio	-0.012* (0.007)	-0.012* (0.007)
Short term debt ratio	0.003 (0.002)	0.003 (0.002)
Debt with interest/invested capital	0.010* (0.006)	0.010** (0.006)
Currency/short term debt	0.074 (0.061)	0.078 (0.061)
Currency/total debt	-0.705*** (0.254)	-0.705*** (0.254)
The open policy of the rating industry		-0.536* (0.278)
Brilliance	0.138 (0.179)	0.147 (0.179)
Lianhe_Fitch	0.327* (0.186)	0.341* (0.186)
Dagong	0.670*** (0.176)	0.692*** (0.176)
Chengxin	-0.178 (0.247)	0.154 (0.247)
Jincheng	0.152 (0.321)	0.149 (0.321)
Lianhe	0.093 (0.246)	0.065 (0.247)
Pengyuan	0.146 (0.168)	0.148 (0.168)
Industry dummies	Yes	Yes
*C*_*1*_	0.894 (0.237)	0.895 (0.237)
*C*_*2*_	4.898 (0.288)	4.903 (0.288)
*C*_*3*_	8.559 (1.028)	8.564 (1.208)

Table 9 reports the numbers in the parentheses are standard errors.

*, **, *** denote that the coefficient is statistically significant at the 10%, 5%, 1% levels respectively.

In the Column 2 model, we include the open policy. The coefficient of the open policy is negative and significant at the 1% level. This result suggests that this open policy truly has a better regulatory effect on preventing rating inflation. Meanwhile, under the open policy of the rating industry, the coefficients of the DRAs and global-partnered CRAs are positive. We can conclude that the open policy leads to rating convergence among DRAs and GRAs in terms of rating upgrades.

Under the open policy, the probability of rating downgrades is 0.880 ($\frac{e^{1.990}}{1+e^{1.990}}$), while the probability of rating upgrades is 0.369 ($\frac{e^{-0.536}}{1+e^{-0.536}}$). The probability of rating downgrades is much higher than the probability of rating upgrades. This result shows that the introduction of the open policy can prevent rating inflation and improve rating quality for DRAs.

To further illustrate the robustness of the results, we utilize the regression results for the model that did not include control variables as the control group. We directly examine the effect of the open policy on rating changes, rating downgrades and rating upgrades.

In Table 10, the coefficients of the open policy, the GRAs and the DRAs are different, but the signs and significance levels are the same. These findings indicate that the empirical tests of the research hypothesis are robust.

**Table 10:** Regression results of no control variables.

Variables	Rating changes	Rating downgrades	Rating upgrades
The open policy of the rating industry	0.378*** (0.024)	2.043*** (0.264)	-0.680*** (0.122)
Brilliance	0.731*** (0.043)	0.405 (0.920)	0.205 (0.175)
Lianhe_Fitch	0.539*** (0.044)	1.395 (0.743)	0.402* (0.181)
Dagong	0.404*** (0.040)	0.650 (0.537)	0.646*** (0.171)
Chengxin	0.040*** (0.045)	0.149 (0.614)	0.209 (0.238)
Jincheng	1.582*** (0.053)	1.250 (0.751)	0.589 (0.320)
Lianhe	0.727*** (0.045)	0.244 (0.522)	0.363 (0.234)
Pengyuan	1.467*** (0.036)	0.678 (0.476)	0.353 (0.162)

Table 10 reports the numbers in the parentheses are standard errors.

*, **, *** denote that the coefficient is statistically significant at the 10%, 5%, 1% levels respectively.

Overall, the empirical results show that the regulatory result of the open policy of the rating industry has better effects on preventing rating inflation and improving the rating quality of DRAs. Furthermore, from the long-term perspective, the open policy leads to rating convergence among the DRAs and the GRAs.

## Conclusion

We research competition among DRAs and GRAs under the open policy of the Chinese rating industry. Based on the analysis, using an improved Hotelling model that considers both the short-term perspective and the long-term perspective, the results suggest that short-term competition among DRAs and GRAs cannot induce DRAs to improve their rating quality and reputation to increase their profits. The entrance of GRAs has an impact on DRAs’ rating business. The open policy of the rating industry has contributed to rating learning among DRAs and global-partnered rating agencies for a long time. The open policy of the rating industry is effective for improving the rating quality of DRAs and GRAs.

In contrast to the previous studies, we conduct an empirical analysis of the effects of the open policy. Our findings demonstrate that the open policy can make rating standards more stringent and that the regulatory result is more effective. In addition, competition among global-partner CRAs and DRAs contributes to higher rating quality under this open policy. More importantly, we analyze the effects of the policy on rating downgrades and rating upgrades. We demonstrate that the open policy of the rating industry leads to rating convergence among DRAs and global-partnered CRAs. According to our research, the open policy of the Chinese industry has a better regulatory effect.

Due to the lack of rating data provided by the GRAs, in this article, we replace GRAs with global-partnered rating agencies. With the establishment of S&P (China), the entry of more GRAs into the Chinese bond market and their development will be of great interest in the future.

## Supporting information

S1 Data (XLSX)

S2 Data (XLSX)
